# Phase-Inversion In Situ Systems: Problems and Prospects of Biomedical Application

**DOI:** 10.3390/pharmaceutics17060750

**Published:** 2025-06-06

**Authors:** Elena O. Bakhrushina, Svetlana A. Titova, Polina S. Sakharova, Olga N. Plakhotnaya, Viktoriya V. Grikh, Alla R. Patalova, Anna V. Gorbacheva, Ivan I. Krasnyuk, Ivan I. Krasnyuk

**Affiliations:** I.M. Sechenov First Moscow State Medical University, Moscow 119991, Russia; bakhrushina_e_o@staff.sechenov.ru (E.O.B.); sakharova_p_s@student.sechenov.ru (P.S.S.); plakhotnaya_o_n@staff.sechenov.ru (O.N.P.); grikh_v_v_1@staff.sechenov.ru (V.V.G.); patalova_a_r@staff.sechenov.ru (A.R.P.); gorbacheva_a_v@staff.sechenov.ru (A.V.G.); krasnyuk_i_i_1@staff.sechenov.ru (I.I.K.J.); krasnyuk_i_i@staff.sechenov.ru (I.I.K.)

**Keywords:** PLGA, solvent exchange, in situ implant, gels, phase-inversion system

## Abstract

Stimuli-sensitive (in situ) drug delivery systems are a dynamically developing area of pharmaceutical research. Over the past decade, the number of studies on such systems has doubled. Among these, phase-inversion (or phase-sensitive) formulations, which were among the earliest proposed, offer significant advantages, including enhanced stability and stimuli-responsiveness. However, phase-inversion systems have remained relatively understudied. Despite the existence of three patented technologies (Atrigel^®^, BEPO^®^, FluidCrystal^®^) for delivery systems utilizing phase inversion for various routes of administration, the absence of unified approaches to development and standardization has significantly impeded the introduction of novel, effective drugs into clinical practice. This review examined the main polymers and solvents used to create phase-inversion compositions and discussed the feasibility of introducing other excipients to modify the systems’ physicochemical properties. The most commonly used polymers included polylactide-co-glycolide, shellac, and polylactic acid. The most frequently used solvents were *N*-methylpyrrolidone and dimethyl sulfoxide. Following an analysis of clinical studies of phase-sensitive drugs conducted over the past 25 years, as well as original research indexed in PubMed, ScienceDirect, and Google Scholar, the main problems hindering the broader adoption of phase-inversion systems in clinical practice were identified, and recommendations for further development in this promising area were provided.

## 1. Introduction

The primary aim of modern pharmaceutical technology is to maximize the therapeutic efficacy of active substances while minimizing systemic adverse effects [1]. One approach to achieving this goal involves the use of in situ systems (ISS)—polymeric compositions that undergo sol–gel transitions in response to physiological conditions [2]. Stimuli-sensitive in situ gels are administered as parenteral dosage forms in various fields, including ophthalmology, vaccine prophylaxis, treatment of neurodegenerative and psychiatric disorders, and oncology [2,3,4,5,6,7]. A rational selection of excipients can enable prolonged release of active substance, facilitate achievement of the optimal value of mucoadhesion, and reduce the invasiveness of injectable dosage forms [8,9,10].

Depending on the nature of the gelation trigger, polymer-based systems are classified as pH-sensitive [11], thermosensitive (including thermoreversible) [12,13], ion-sensitive [14], photosensitive [15], and phase-inversion systems [16]. In 2020, cutting-edge studies identified two additional promising stimuli—redox potential and the presence of specific enzymes—which offered high targeting potential for intratumoral implantation of ISS [17].

The first references to ISS in the scientific literature date back to 1987 and describe phase-inversion compositions; the first patent was filed in 1990 [18]. Despite being the earliest developed ISS, phase-inversion systems have remained the least explored within the global scientific community. According to Google Scholar, the total number of publications (including patents) for different ISS types was as follows: pH-sensitive systems—98,500; ion-sensitive—54,400; thermosensitive—60,800; photosensitive—64,900; and phase-inversion systems—only 5010. Data from the ScienceDirect database indicated that, for over 20 years, no more than two articles per year had been published on this topic—until a resurgence of interest began in 2021.

In the scientific literature, systems that form at the site of administration due to solvent diffusion from the injected pharmaceutical composition into the surrounding soft tissue are referred to by various terms. The most commonly used designations are “phase-sensitive” (17%) and “phase-inversion” (76%) in situ systems, while the term “solvent exchange-induced in situ-forming gel” is used less frequently (7%). Notably, the variation in terminology—and the absence of a universally accepted name within the scientific community (unlike many other ISS)—hinders the harmonization of research findings and the development of phase-inversion systems. In this study, the key characteristics of phase-inversion systems were reviewed, their promising applications as in situ implants were explored, and potential reasons for their limited usage in clinical practice were discussed. During a large-scale analysis of international scientific literature, we found a lack of reviews specifically focusing on phase-inversion technology, or other ISS employing this mechanism. Furthermore, this review is the first to comprehensively examine clinical studies related to these systems and to perform a SWOT analysis. Therefore, this review is the first to describe all aspects of phase-inversion in situ systems.

## 2. Basic Characteristics of Phase-Inversion Systems

In this section, the mechanism of action, advantages and disadvantages of phase-inversion ISS for medical application were reviewed, as well as the most commonly used excipients and their characteristics.

### 2.1. Advantages and Disadvantages

**Figure 1:**
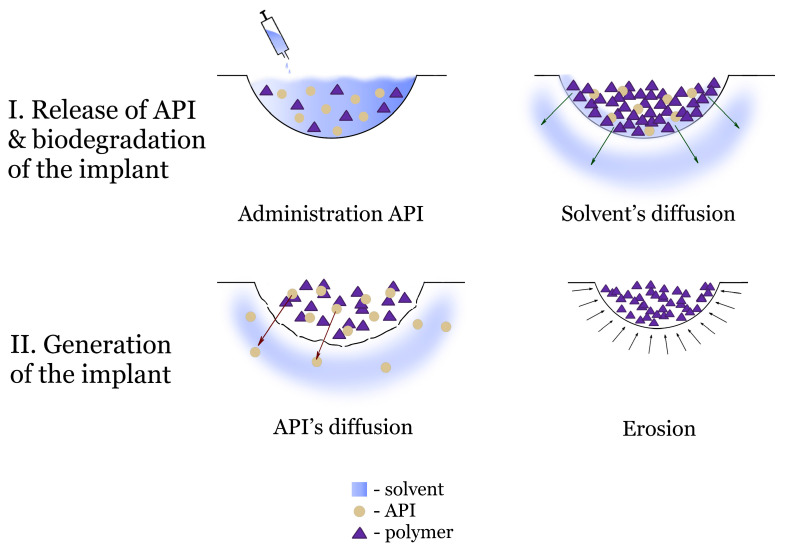
Mechanism of phase-inversion implants.

Phase-inversion ISS are composed of a polymer and an organic solvent. The phase transition mechanism of phase-inversion in situ implants is based on liquid–liquid extraction at the injection site [19]. Among the in situ forms created by solvent diffusion into surrounding soft tissues, in situ implants are the most well known. These are solid polymeric scaffolds formed after the solvent had dissipated from the composition, capable of releasing the active ingredient—initially through diffusion, followed by matrix erosion (Figure 1).

Such systems may also be used for their mechanical functions. For example, in dental practice, a phase-sensitive ISS can be employed soft tissue fusion, thereby maintaining the surgical site in preparation for further implantation. Depending on the composition and rate of solvent exchange, systems with lower mechanical strength—effectively in situ gels—could also be formed. These softer, pseudoplastic compositions are considered particularly suitable for chemoembolization or subcutaneous implantation.

**Figure 2:**
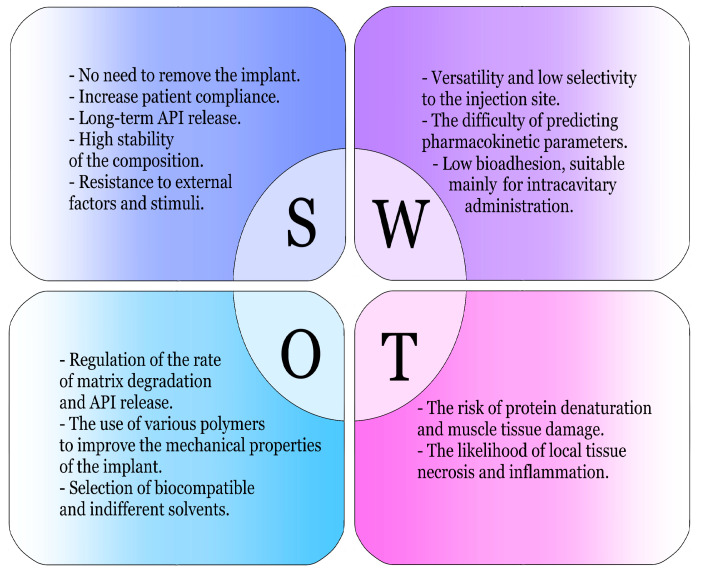
SWOT analysis of the application of phase-inversion ISS [20,21,22,23,24,25,26].

The main advantages and disadvantages of phase-inversion systems are summarized in Figure 2.

### 2.2. Main Matrix Formers

To fully realize the benefits of phase-inversion systems, the components of the composition are required to meet several key criteria. Matrix-forming polymers should be biodegradable, non-toxic, and possess appropriate viscosity and mechanical strength for specific clinical applications [27]. Currently, polymers such as lactic acid homopolymer, polylactide, polycaprolactone, *N*-lauroyl-L-alanine methyl ester, sucrose acetate isobutyrate, borneol, and PLGA (polylactide-co-glycolide) are considered suitable for this purpose. Among these, PLGA accounts for approximately 65–67% of studies in the field [28]. An overview of the current status of these matrix formers is provided in Table 1.

#### 2.2.1. Lactic Acid Homopolymer

Lactic acid homopolymer is FDA-approved [30,34], biocompatible, biodegradable, and exhibits low immunogenicity [35,36]. It is characterized as a strong, thermoplastic, rigid polymer with a glass transition temperature above 58 °C [37,38], making it suitable for use in regenerative medicine [39]. Notably, it demonstrates high encapsulation capacity and can undergo various chemical modifications. However, a major drawback is the characteristic “explosive” initial release, commonly observed in phase-inversion implants [40]. Additionally, it has a relatively long degradation period and a limited capacity to incorporate hydrophilic substances [41].

#### 2.2.2. Polycaprolactone

Polycaprolactone (PCL) is classified as a biocompatible aliphatic synthetic polymer with a melting point of 59–64 °C and a glass transition temperature of −60 °C [42]. Depending on the production technology, its porosity can range from 15% to 70% [43]. PCL has been shown to be miscible with a wide range of substances, including other polymers and low-molecular-weight compounds [44]. It exhibits favorable viscoelastic properties, but biodegrades more slowly than other polymers—often taking up to two years [45,46]. This has been attributed to its high crystallinity and hydrophobicity, which can be altered using acryloyl chloride modification [47].

Importantly, polycaprolactone is commercially available and widely used in medical applications, from suture materials to bone tissue engineering [48]. Recent research also has highlighted its potential for use in vascular grafts and assisted reproductive technologies [49,50]. However, it should be noted that some studies have reported cytotoxic and ecotoxic effects associated with biomedical-grade polycaprolactone [51].

#### 2.2.3. *N*-Lauroyl-L-alanine Methyl Ester

**Figure 3:**
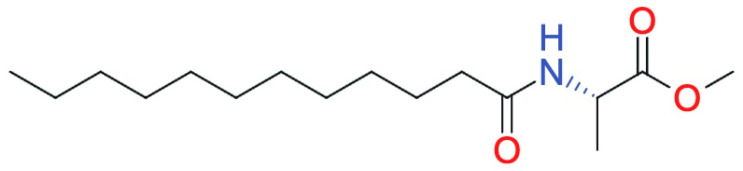
*N*-lauroyl-L-alanine methyl ester formula (Reproduced/adapted from [56]).

The application of this compound in biomedicine has been underrepresented in the international scientific literature. This may be attributed to the fact that in situ gelation of *N*-lauroyl-L-alanine methyl ester requires relatively high concentrations (approximately 30%) and a prolonged gelation time (up to 110 min) [52]. Despite these limitations, existing studies have described this matrix-forming agent as biocompatible, capable of high drug loading (up to 86.05%), and both thermo- and redox-sensitive [53]. Notably, the sol–gel transition can be modulated by adjusting the concentration, enabling the development of compositions that gel at physiological temperatures [54]. Due to its amphiphilic structure, this ester has been shown to incorporate both hydrophilic and hydrophobic active pharmaceutical ingredients (Figure 3) [55].

Considering these features, a potentially more promising technological application of *N*-lauroyl-L-alanine methyl ester was proposed to be its use in nanoparticle stabilization [57].

#### 2.2.4. Sucrose Acetate Isobutyrate

**Figure 4:**
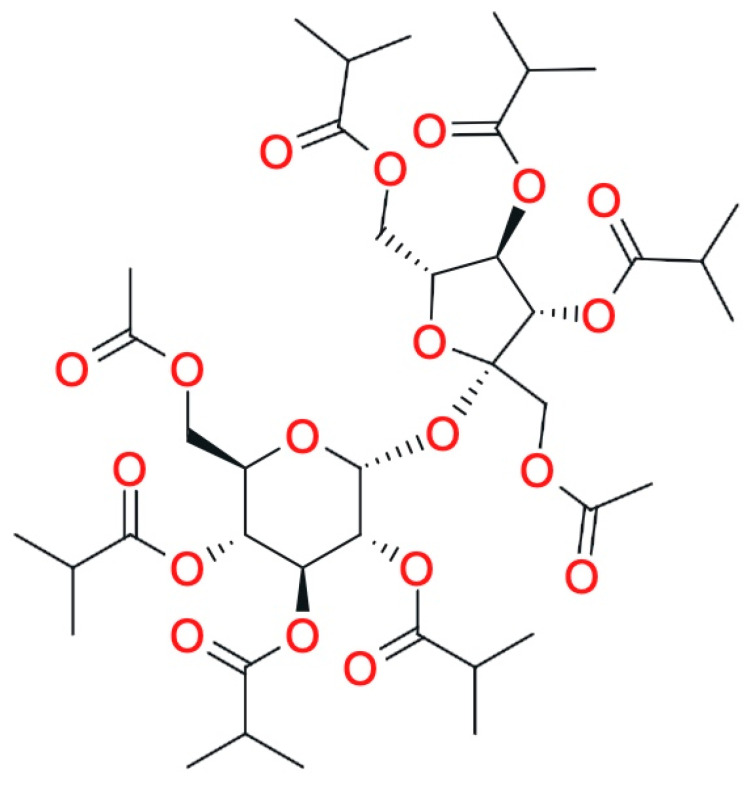
Sucrose acetate isobutyrate formula (Reproduced/adapted from [60]).

Sucrose acetate isobutyrate (SAIB) is classified as a biocompatible, highly viscous matrix-forming agent derived from the esterification of sucrose with acetic and isobutyric anhydrides (Figure 4) [58,59].

In in vitro experiments, this polymer was demonstrated to enhance the stability and pharmacokinetic profiles of Biopharmaceutics Classification System (BCS) class II and IV substances [61]. More recently, SAIB was investigated as a long-acting delivery vehicle for steroid hormones [62]. In studies involving CACO-2 cell cultures, it exhibited mucoadhesive properties [63]. Notably, the incorporation of SAIB into formulations with other matrix formers was found to prolong active ingredient release by up to 4.7 times compared to the use of individual components, while also improving elasticity and reducing brittleness [64,65]. Furthermore, SAIB-containing compositions have demonstrated the lowest initial “explosive” release among components used in phase-sensitive ISS [66].

#### 2.2.5. Borneol

**Figure 5:**
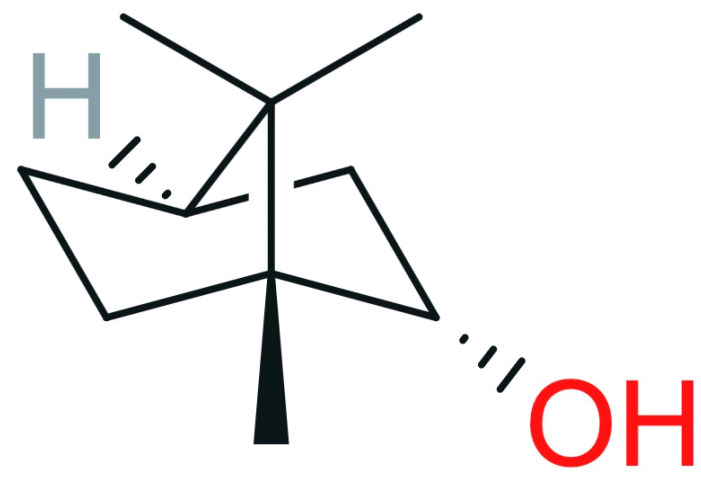
Borneol formula (reproduced/adapted from [68]).

Borneol is identified as a bicyclic monoterpene alcohol (Figure 5) [67].

This terpenoid serves both as a pharmacologically active compound and an excipient. Evidence suggests that borneol might mitigate exogenous oxidative stress and exhibit potential DNA-protective properties [69,70]. As an adjuvant, borneol was shown to enhance the permeability of physiological barriers [71,72], with statistically significant results demonstrated in compositions containing ribavirin, ofloxacin, and tobramycin [73]. Studies on fibroblast cultures reported minimal cytotoxicity and mild bacteriostatic activity [74,75]. However, its application as a matrix-forming agent is constrained by the requirement for dimethyl sulfoxide as a solvent to achieve an effective phase transition [76].

#### 2.2.6. Shellac

Shellac is recognized as a natural, biocompatible, and biodegradable resin [77]. Its amphiphilic structure imparts surfactant properties and the capacity to form complexes with other polymers [78]. Shellac’s solubility was found to be pH-dependent, granting it potential as a pH-sensitive gelator and enabling its application in dual stimulus-sensitive systems [79,80]. However, one limitation was identified as the “aging” phenomenon, characterized by decreased flexibility and in-creased brittleness over time, which adversely affected composition stability [81,82]. This issue was reported to be partially mitigated by incorporating other biocompatible polymers [83].

#### 2.2.7. Polylactide-Co-Glycolide

**Figure 6:**
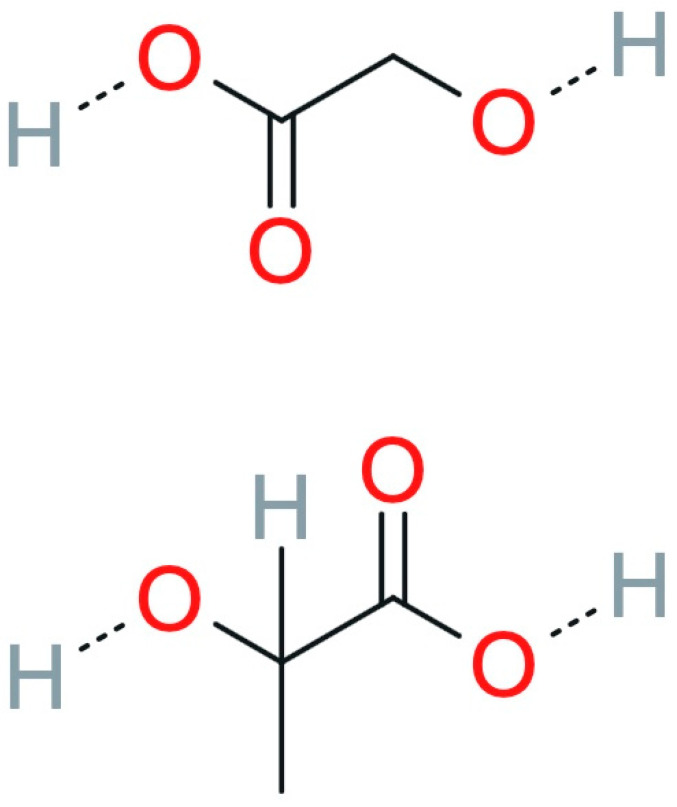
PLGA formula (reproduced/adapted from [85]).

Polylactide-co-glycolide (PLGA) is described as a biocompatible and biodegradable copolymer composed of lactic and glycolic acid, synthesized in a wide range of monomer ratios (Figure 6) [84].

Characteristics such as end functional groups, polymer molecular weight, and monomer ratio were found to play a decisive role in determining the swelling kinetics of PLGA and the release rate of the active substance from the implant [86]. The degradation rate was shown to be slowed by polyethylene glycol (PEG) modification [87] and accelerated by the addition of poloxamer 188 [88]. Release kinetics were further modulated by altering the implant size [89]. Thus, PLGA exhibits tunable biodegradability [90]. Although these findings were based on in vitro studies, extrapolation from tissue-engineered implant data suggested that, under in vivo conditions, drug release kinetics might extend to 8–16 weeks [84,91].

Preliminary conclusions can be drawn regarding the limited prevalence of phase-inversion ISS in contemporary pharmaceutical research. Notably, the most common matrix formers were found to be largely absent from the pharmacopeias of various countries. Despite some compounds having received FDA approval, they were not yet recognized universally as suitable for all intended applications of phase-inversion systems. This limitation could likely be attributed to the unique characteristics of these systems, which pose challenges for standardization and practical implementation in healthcare.

Moreover, the relatively high cost of materials employed in these systems could be identified as a significant barrier to their broader adoption. Attempts to reduce expenses by synthesizing modified polymers are often associated with decreased stability of biocompatible matrix formers during storage. For example, a study by K. Schoenhammer et al. investigated the PEGylation (modification with polyethylene glycol) of PLGA, demonstrating that the main factor contributing to instability was hydrolysis of the copolymer, even at trace water levels (1–1.12%). Moreover, reactive functional groups were reported to interact with nucleophilic solvent fragments and active pharmaceutical ingredients, further compromising stability [90]. On the other hand, a 12-month cost-minimization analysis was conducted in nine European countries by Retsa M. et al., using leuprolide acetate-based phase-sensitive depot compositions, showing that these systems were economically justified. Among formulations with comparable efficacy, those with the longest release duration resulted in the lowest healthcare costs [91]. However, whether these findings can be extrapolated to therapeutic areas with inherently lower treatment costs than oncology remains uncertain.

### 2.3. Main Solvents

Solvents used in phase-sensitive systems are expected to meet several critical requirements as well, including low toxicity, effective solubilizing capacity, and appropriate diffusion rate and extent into surrounding tissues [92]. A wide range of organic solvents—used individually or in combination—was found to satisfy these criteria. The most widely studied solvents are reviewed below, with a summary of their current regulatory and experimental status provided in Table 2.

#### 2.3.1. *N*-Methylpyrrolidone and 2-Pyrrolidone

The application of 2-pyrrolidone in medical and pharmaceutical research has remained relatively underexplored. Nonetheless, it exhibits superior solubilizing properties compared to many other organic solvents [93]. Greater attention has been given to its substituted derivatives, particularly *N*-methylpyrrolidone (NMP) [94,95].

NMP is identified as a five-membered lactam known for its high polarity, low viscosity, and low volatility [96,97]. In addition to being used as a solvent or co-solvent [98,99], and as a skin permeation enhancer [100,101], NMP has demonstrated intrinsic pharmacological effects. For instance, in vitro studies have suggested that NMP can inhibit osteoclast activity, potentially mitigating bone resorption in osteoporosis [102].

However, the use of NMP has been limited by its relatively high environmental hazard [103], inconclusive reproductive toxicity data from in vivo studies [104,105,106], and its potential to cause mucosal irritation in humans [107].

#### 2.3.2. Dimethyl Sulfoxide

Dimethyl sulfoxide (DMSO) is recognized as a polar, amphiphilic solvent [108] widely used for enhancing the permeability of active pharmaceutical ingredients across biological barriers and as a cryoprotectant in cell and stem cell preservation [109,110]. It has been approved by the FDA for both topical use and administration into body cavities [111]. Nevertheless, DMSO use has been associated with adverse reactions such as hyponatremia, fever, and bradycardia [112].

Pharmacological activity has also been observed in DMSO, including anti-inflammatory effects, protection against ischemic damage [113], and reduction in collagen degradation [114,115]. These properties have been attributed to its interactions with lipid membranes and ion channels [116], and cytotoxicity has not been demonstrated in relevant in vitro models [117]. However, potential nephrotoxicity, hepatotoxicity, teratogenicity, and mucosal or respiratory tract irritation remain significant concerns [118].

#### 2.3.3. Benzyl Benzoate and Benzyl Alcohol

Benzyl benzoate and benzyl alcohol are classified as clear, aromatic organic liquids with good miscibility across many solvents, allowing concentration adjustments to tailor their effects [119,120]. Both have been commonly used in various industries, including pharmaceutical [121,122]. Their popularity has stemmed from effective solubilizing properties and the preservative action of benzyl alcohol [123,124,125]. Additionally, these solvents have been used as active pharmaceutical ingredients in the treatment of ectoparasitic infections [126,127], and estrogen-like effects have been reported [128]. It is important to note that benzyl benzoate carries a risk of severe adverse effects, including potentially fatal toxicity in newborns [129]. Nonetheless, extensive toxicological evaluations have not revealed significant genotoxic, reproductive, or sensitizing risks [130].

Emerging literature has also reported regenerative [131] and anesthetic [132] properties of benzyl alcohol. Its ability to increase the pathogenicity of *Staphylococcus aureus* has also been established [133]. Studies mention the cytotoxicity of benzyl alcohol for human and animal cells even at low doses [125]. Though toxicity has remained rare in clinical use, it can lead to multi-organ dysfunction [134]. From a composition perspective, benzyl alcohol has been shown to dissolve PLGA only within a narrow lactic-to-glycolic acid ratio range [135].

#### 2.3.4. Triacetin and Triethyl Citrate

Triacetin (glycerol triacetate) has been predominantly used in pharmaceutical formulations as a plasticizer [136] and co-solvent [137], particularly in polymer chemistry [138]. It has also been used in antifungal treatments [139]. In vivo studies have indicated that triacetin may promote weight loss and enhance protein metabolism without negatively affecting mineral metabolism when administered parenterally [140,141,142,143]. Evidence has supported its potential in overcoming chemoresistance in brain tumors when combined with chemotherapeutics [144]. Notably, no acute or subchronic toxicity or environmental hazard data have been reported for triacetin [145].

Triethyl citrate has been recognized as another highly effective [146,147] biocompatible [148] plasticizer, notable for its strong mucoadhesive properties [149]. Unlike triacetin, in vitro studies have suggested that triethyl citrate may enhance lipogenesis [150]. Toxic effects have been documented at high doses, including hypocalcemia [151], mitochondrial dysfunction [152], and potential myotoxicity [153].

#### 2.3.5. Glycofurole

Glycofurole is characterized as a versatile solvent compatible with a broad range of active substances, enhancing their absorption while maintaining a low risk of adverse reactions [154,155]. Its low viscosity and slow diffusion at the injection site make it suitable for injectable formulations [156,157]. Glycofurole has been shown to increase drug loading [158,159] and improve bioavailability in subcutaneous applications during in vivo studies [160], although concerns have been raised regarding its potential to alter hepatic microsomal metabolism [161].

#### 2.3.6. Ethyl Lactate

Ethyl lactate, an FDA-approved solvent [162] and plasticizer [163], is considered environmentally safe and biocompatible, as it is metabolized into endogenous compounds [164,165]. It facilitates uniform drug distribution in polymeric systems and accelerates absorption through mucosal membranes [166,167]. Due to these properties, it has been regarded as a promising candidate for medical applications [168].

#### 2.3.7. Propylene Carbonate

Propylene carbonate is classified as a polar, aprotic solvent that enables efficient drug incorporation into polymers and supports prolonged release [169]. It satisfies core criteria for use in pharmaceutical excipients and in situ depot formulations. Its high stability, water miscibility, low toxicity [170], and ability to dissolve PLGA (as the most popular polymer for the production of phase-inversion implants) across various lactic–glycolic ratios [135] make it particularly attractive. Nevertheless, further toxicological evaluation has been deemed necessary to confirm its safety profile [171].

The regulatory and experimental status of solvents in phase-sensitive systems mirrors the situation observed with matrix-forming agents. While some solvents—such as NMP, DMSO, triacetin, and triethyl citrate—have accumulated sufficient data to support their near-future adoption in clinical practice, while others require further investigation.

It is crucial to recognize that the optimal selection of a matrix-forming agent and solvent alone does not guarantee effective formulation. The interaction between these components, as well as their relative proportions, has been shown to significantly influence implant morphology, in situ formation kinetics, and drug release profiles—factors confirmed across in vitro, ex vivo, and in vivo models [172]. Researchers have pointed to hydrogen bonding and temperature-dependent sol–gel transitions of certain copolymers as contributing mechanisms to these outcomes [173].

## 3. Application of Phase-Inversion Systems

In this section, both registered pharmaceutical products and current developments in phase-inversion systems were reviewed, with discussion of their potential, current applications, and limitations.

### 3.1. Existing Preparations and Prospective Modifications

The use of the aforementioned excipients has been well established in contemporary clinical practice. However, their application in the form of phase-inversion systems has remained limited to a narrow range of compositions, primarily those produced using the patented Atrigel^®^ technology. According to a literature review conducted by Anjali Jain et al., Atrigel^®^ might be considered almost universal. Notably, the authors highlighted the flexibility of this technology, which permitted the use of both hydrophilic and hydrophobic solvents and matrix-forming agents, thereby enabling its adaptation to a broad spectrum of active pharmaceutical ingredients (APIs) [174].

The dental preparation Atridox^®^ has gained traction in clinical use. This composition consists of a tetracycline antibiotic embedded within a polylactide matrix-former and NMP as a solvent, delivered via a dual-syringe system [174,175]. A meta-analysis demonstrated that Atridox^®^ exerted therapeutic effects not only through its active ingredient but also by alleviating periodontal inflammation and gingival bleeding [176], even in cases with complex dental histories where alternative treatments had proven ineffective [177]. This product has been recognized not only for its clinical efficacy [178] but also as a cost-effective alternative to conventional depo-formulations, due to its user-friendly application and modifiable manufacturing process [179]. Atrisorb^®^, a similar preparation, shares comparable mechanisms of action, therapeutic indications, and composition [174].

In addition to topical applications, phase-inversion systems have also been employed in formulations with systemic effects. One notable example is the depot formulation of leuprolide acetate, marketed as ELIGARD^®^ [180], intended for the treatment of advanced hormone-dependent prostate cancer [181,182]. ELIGARD^®^ is notable for offering prolonged-release formulations that maintain therapeutic efficacy for one to six months [183,184], and for its favorable tolerability profile compared to standard therapies [185,186,187]. Some literature has positioned this drug as a reference therapy within its indication [188], citing its comparable efficacy to bilateral orchidectomy [189]. Nevertheless, its administration remains relatively complex—more labor-intensive than the insertion of traditional implants and not yet simplified to the degree achieved by newer compositions [190].

One such advancement is BEPO^®^, an in situ-forming depot system comprising a polymer blend with controlled release kinetics, validated through in vivo studies across multiple drugs [191,192]. Morphological properties of the resulting depot can be modulated by adjusting the solvent diffusion rate, as demonstrated by F. Ng et al. in both in vitro and in vivo settings. Another key feature of BEPO^®^ is the ability to tune biodegradation kinetics via polymer composition, confirmed using nuclear magnetic resonance (NMR) and gel chromatography analysis [193]. Importantly, long-term in vivo studies reported no adverse reactions associated with subcutaneous administration when di- and tri-block PEG-PLGA copolymers were used, as supported by histological assessments [194]. Additionally, an in vivo study involving ivermectin in cattle found no statistically significant effect of matrix-former composition on efficacy, further supporting the versatility of this technology [195].

Another technology inspired by ELIGARD^®^ is FluidCrystal^®^ [190]. Although its mechanism and release profile are similar to those of traditional phase-inversion systems, FluidCrystal^®^ employs a smaller quantity of organic solvents and biocompatible lipid-derived matrix formers [196]. To date, this approach has been most extensively studied with buprenorphine [197,198,199], octreotide [200], and pasireotide [201].

Over the past 25 years, numerous clinical trials have evaluated drugs employing phase-inversion technologies. As part of this analysis, registered clinical trials listed in the ClinicalTrials.gov and EudraCT databases, as well as relevant retrospective studies and Supplementary Materials from peer-reviewed journals, were reviewed. A total of 29 relevant studies were identified: 17 (58.62%) investigated Atrigel^®^, 2 (6.9%) studied BEPO^®^, and 9 (31.03%) focused on FluidCrystal^®^. A detailed analysis of specific polymers, solvents, or associated terms (e.g., “in situ gel-forming”) was hindered by inconsistent terminology in trial descriptions, highlighting a broader issue in the standardization of nomenclature within this field.

Among the clinical trials involving Atrigel^®^, the most common study design was a multicenter and open-label study (35.29%). Only three studies were placebo-controlled (17.65%), including one double-blind trial (5.88%). The first reported clinical trial data for a phase-inversion system were related to Atridox^®^ (2003–2009). Although detailed results were not publicly released, this study marked an early milestone in the clinical validation of phase-inversion systems. Regulatory requirements were less stringent for topical formulations, making safety considerations less critical for Atridox^®^ (ADX) compared to Eligard^®^ (LPA), which was approved by the FDA four years later [186].

Since the approval of Atrigel^®^ technology in 2002, the number of associated clinical trials has steadily increased. These studies included injectable APIs such as buprenorphine (BPN) and risperidone (RPD), likely selected to address the need for long-acting preparations that maintain consistent therapeutic concentrations, reduce frequency of administration, minimize side effects, and enhance patient compliance.

Clinical trials of drugs manufactured using BEPO^®^ technology began in 2018, with one Phase 3 trial currently ongoing and expected to conclude in 2027. The API selected for investigation was celecoxib (CLX), a nonsteroidal anti-inflammatory drug, tested in comparison with standard pain management regimens. As of yet, the results have not been published.

FluidCrystal^®^ technology has featured more prominently in clinical trial registries over the past 15 years, possibly due to the established biocompatibility of its components. A broader range of APIs has been tested using this technology, including buprenorphine (BPN), leuprolide acetate (LPA), octreotide (OCT), and pasireotide (PST). These studies often employed randomized multicenter designs, and double-blind trials have also been conducted. A notable feature of this research has been the frequent inclusion of comparison products—such as films or injectables with different API release rates.

Key parameters from the reviewed clinical trials on phase-inversion systems are summarized in Table 3.

Based on the above data, several conclusions were drawn:

(1) Buprenorphine (BPN) Studies within the Atrigel^®^ System:

Clinical trials involving BPN formulated using the Atrigel^®^ system revealed several limitations. Most notably, the absence of comparison drugs in the majority of studies precluded definitive conclusions regarding the influence of this delivery system on the safety and efficacy profiles of the active pharmaceutical substance. Furthermore, only two studies employed a placebo control [208,209,210], likely due to the ethical complexities associated with placebo use in opioid dependence therapy. Additionally, the inherent challenges of treating opioid dependence—such as its multifactorial etiology, frequent comorbidities, and the strong influence of patients’ subjective experiences—complicated data interpretation.

In contrast, studies evaluating BPN within the FluidCrystal^®^ system provided more comprehensive data. For example, comparative analysis with comparison drugs: sublingual tablets, infusion solutions, and placebo plus buprenorphine/naloxone have demonstrated fewer adverse effects [200] and significantly higher efficacy [233] for the FluidCrystal^®^ BPN formulation.

(2) Risperidone (RPD) in Atrigel^®^ Formulation:

More robust data was available for RPD as part of the Atrigel^®^ system. Studies have confirmed its influence on pharmacokinetic parameters when transitioning from oral to subcutaneous administration [217]. Reported side effect rates were generally comparable to those of placebo (e.g., 70.4% and 77.8% for two treatment groups vs. 68.6% for placebo) and in some cases, even lower (e.g., extrapyramidal symptoms occurred in 7.8% of patients at the 90 mg dose vs. 9.3% for placebo) [219]. Nevertheless, further comparative studies with conventional dosage forms are recommended to substantiate these findings.

(3) Celecoxib (CLX) in the BEPO^®^ System:

At present, the available data on celecoxib delivered via the BEPO^®^ system was considered insufficient to conduct a thorough assessment of its safety and efficacy.

(4) FluidCrystal^®^ Formulations of Octreotide (OCT) and Pasireotide (PST):

Studies evaluating FluidCrystal^®^ OCT and PST formulations were among the most detailed and provided significant insight. Two clinical trials compared FluidCrystal^®^ OCT with a conventional long-acting octreotide formulation. These studies reported a higher incidence of adverse events with the FluidCrystal^®^ version, with the exception of headache, despite equivalent dosing. Notably, IGF-1 suppression was superior with FluidCrystal^®^ OCT only at the first injection and, in some groups, at the third injection [200], findings partially contradicted earlier studies conducted in smaller patient cohorts [236].

In case of FluidCrystal^®^ PST, the results were more favorable. For instance, the 5 mg dose exhibited a lower incidence of adverse drug reactions compared to both extended- and immediate-release forms. For other dosages, overall tolerability was superior to the immediate-release form (except for local reactions) but inferior to the prolonged-release form. Optimal tolerability was observed with gluteal administration, although this conclusion is limited by the exclusive study of the 20 mg dose [201].

Based on these findings, it can be assumed that in certain contexts, in situ-forming systems (ISS) could serve as viable alternatives to conventional long-acting dosage forms. However, the feasibility of this substitution must be evaluated individually for each active substance.

(5) Comparison of Eligard^®^ and FluidCrystal^®^ Leuprolide Acetate (LPA):

Although a comparative study between Eligard^®^ and FluidCrystal^®^ LPA has been registered, the results are not currently available in the public domain, thereby limiting direct comparisons between these technologies [237].

Thus, the number of clinical trials investigating phase-inversion systems is steadily increasing, reflecting progress in overcoming regulatory barriers. However, further comparative studies are warranted, alongside the efforts to enhance data accessibility for the global scientific community.

### 3.2. Application in Dental Practice

The field of dentistry has generated the highest volume of publications concerning the application of phase-inversion systems, particularly in the management of periodontitis [238]. The popularity of these systems in dental applications might be attributed to their pronounced local effects, adequate mucoadhesive properties, structural stability, and ability to conform precisely to periodontal pockets. This was corroborated by a 2019 study by Fareeha Batool et al., which employed human oral epithelial cells, *P. gingivalis* cultures, and in vivo models. Another reason demonstrated by researchers was the fact that therapy for periodontitis allowed the usage of several pharmacological groups at once, from antibacterial and antiseptic agents to non-steroidal anti-inflammatory drugs [239].

An additional advantage of dental use is the broader acceptability of excipients not commonly used in injectable formulations for systemic administration (intravenous, intramuscular, or subcutaneous). This expands the range of compatible polymers and allows the inclusion of novel plasticizers that may enhance adhesion and synergistic effects [240,241,242,243].

Several implant modifications have been proposed to enhance anesthetic effects and replace toxic solvents like NMP. For example, F. Ramos et al. proposed alternative solvent mixtures comprising polyethylene glycol 400, triethyl citrate, and ethanol in ratios of 85:10:5 and 60:30:10, using chlorhexidine hydrochloride and ibuprofen as model drugs. The study assessed needle permeability, antibacterial activity against *Streptococcus parasanguinis*, and in vitro release rates. Although cytotoxicity or in vivo adverse effect profiles were not evaluated, the independent biocompatibility of the proposed excipients supports their potential for future investigation [244].

Another promising direction involves enhancing antibacterial efficacy and co-loading multiple active substances. Such combinations could address polymicrobial oral infections, promote bone regeneration, and exert localized immunomodulatory effects [245]. However, limitations exist regarding the choice of matrix formers. For instance, compositions based on rosin exhibited minimal antibacterial activity against common periodontal pathogens—including *Staphylococcus aureus*, *Porphyromonas gingivalis*, *Candida albicans*, and *Streptococcus mutans*—compared to those containing propolis [246].

At the same time, it should be noted that the relative release rate parameters of both anti-inflammatory and antibacterial agents in such compositions cannot depend solely on the composition itself, but vary significantly depending on the chemical nature of the drug. Moreover, these parameters cannot be fully explained by correlations with the value of relative concentration or solubility, highlighting the need for further research [247]. Despite this observation, no reduction in the bactericidal effect was observed against *Streptococcus sanguinis*, *S. constellatus*, *Porphyromonas gingivalis*, *Actinomyces odontolyticus*, *Fusobacterium nucleatum*, *F. naviforme*, *Veillonella dispar*, and *Prevotella nigrescens* when using combined compositions that included a plasticizer and an adhesive component [248].

Another important feature should be emphasized: when using phase-inversion systems, it was possible to achieve active-substance release—typically an antibiotic—over a period ranging from 10 h to one or two weeks, depending on the composition base, with matrix degradation occurring within approximately one month. This imposed certain limitations on expanding the practical applications of these compositions [249,250,251]. Nevertheless, the popularity of phase-inversion systems in dental practice is attributed to their capacity to modulate biodegradation rate and pharmacokinetic parameters within defined limits, accommodate a broad range of active pharmaceutical substances, reduce systemic adverse reactions, improve patient adherence to treatment, and potentially contribute pharmacological activity from matrix components themselves [252,253,254].

### 3.3. Oncology

The availability of a registered drug product in this domain has made oncology the second most prominent area for the development of phase-inversion systems. One major challenge in oncology is managing the severe unwanted reactions associated with many anticancer therapies. Intratumoral administration of depo-forms has been proposed as a promising strategy to mitigate systemic toxicity. Sequential in vitro and in vivo studies using a murine tumor model demonstrated a significant reduction in chemotherapy-related toxicity (specifically for doxorubicin) and an increase in survival compared to the control group [255].

However, the use of some potentially effective substances is limited by their physicochemical properties. Recent in ovo studies evaluating the incorporation of such agents into polymer matrices have shown promise—not only enabling prolonged release and stable bioavailability, but also suggesting potential synergistic effects with standard chemotherapeutics, which were not achievable via oral administration [256].

The literature also describes complex compositions responsive to multiple physiological stimuli, including molecular ones. For instance, a biocompatible and biodegradable nitric oxide-modified polymer has been developed to exert targeted immunomodulatory effects within the tumor microenvironment. This system was evaluated for biocompatibility using BMDM cell cultures, with successful endocytosis demonstrated in 4T1 cells, which were also used to establish a mouse tumor model [257]. However, the matrix-forming agents in this system consisted of poorly studied complex polymers, making it difficult to predict their efficacy in human applications, despite their conceptual alignment with phase-sensitive systems.

Another direction of development involves depo-forms, where the phase transition is carried out using completely new compositions. Ethanol, for example, was investigated as a solvent (though not suitable for PLGA-based systems [135]), with phospholipids replacing conventional polymers [258]. This system enabled efficient incorporation of protein, lipid, and various chemotherapeutic drugs while minimizing the risk of serious adverse reactions [259]. A relatively short duration of local therapeutic effect remained the primary limitation [260]. This underscores the need for continued research into pharmacokinetic modulation—particularly as improved tolerability is often prioritized over dosing frequency in oncology [261]. In some cases, this limitation might be addressed by direct intratumoral injection [262], though this approach was challenged by high interstitial fluid pressure [263] or required adjunctive non-invasive manipulations [264].

### 3.4. Other Areas of Application

Currently, the broader medical adoption of phase-inversion systems is largely constrained by limited experimental data and requires further research. Nonetheless, for certain nosologies discussed below, available findings clearly indicated strong potential for further development.

#### 3.4.1. Therapy of Oropharyngeal Candidiasis

The success of phase-inversion systems in local antimicrobial therapy has spurred exploration into their antifungal applications. Jongjan Mahadlek et al. proposed an innovative system using lauric acid as a matrix-forming agent and NMP as a solvent. The system met the criteria for phase-sensitive gels, including appropriate pH and gelation rate in phosphate buffer. The clove oil-based active ingredient demonstrated antimicrobial activity against six bacterial species, with the strongest effect observed against *Candida albicans* [265].

Fungal infections often present therapeutic challenges—systemic unwanted reactions from oral drugs and insufficient efficacy of topical treatments, especially in complex anatomical regions like the oropharynx [266]. In response, Mahadlek et al. (2022) developed a sprayable phase-inversion system containing lauric acid and clotrimazole that formed an adhesive gel-film under physiological conditions. While the system demonstrated favorable pH and mucoadhesion in ex vivo studies, higher lauric acid content (from a 20:20 to 25:25 ratio) accelerated gelation but increased viscosity, as confirmed via rheometry—thereby compromising sprayability [267]. Notably, this study focused only on parameters of the system without evaluating its therapeutic efficacy.

#### 3.4.2. Musculoskeletal Diseases

In recent years, interest has grown in applying phase-inversion ISS in tissue engineering and musculoskeletal diseases [268,269], conditions often characterized by chronic pain, aseptic inflammation, or bacterial infection [270,271]. Federico Karp et al. developed a combination of PLGA and Eudragit matrix formers with different compositions using florfenicol as a model drug. The composition demonstrated activity against Pseudomonas aeruginosa, a common pathogen in trauma-associated infections. Through a combination of electron microscopy, FTIR spectroscopy, release profiling in phosphate buffer, and mathematical modeling, a formulation containing Eudragit E100 achieved optimal active ingredient encapsulation, avoided “burst release”, and maintained antibacterial effect for 12 days [268].

Long-acting analgesics represent another promising application. Ziyi Yang et al. (2024) reported a PLGA/NMP system containing meloxicam, a non-steroidal anti-inflammatory drug. Comprehensive evaluation included implant morphology, drug loading efficiency, and analgesic efficacy in a murine hot plate test, all yielding promising results [272].

Beyond symptom relief, in situ implants for intra-articular injection of disease-modifying drugs are under development. PLGA matrices addressed a key challenge—rapid lymphatic clearance of intra-articular drugs—allowing for reduced dosing frequency and drug volume while maintaining efficacy in in vivo studies [273,274,275].

#### 3.4.3. Future Application Perspectives

While studies have proposed the use of phase-inversion systems for common diseases, many of these have yet to reach clinical use or gain widespread scientific traction. For example, insulin depot-forms with release durations of 3 to 60 days have been explored [276], but complex release kinetics and “burst release” remain the main challenges. Lexi Wang et al. addressed this by modifying the polymer-to-solvent ratio to 1:4 and incorporating 50% of the active ingredient, achieving uniform release and faster in vitro gelation without affecting rheological properties [277]. In vivo studies using phospholipid-based matrices also indicated stable prolonged release [278].

Similar approaches may be applied to other hypoglycemics, such as sulfonylurea derivatives. While PLGA compatibility with glibenclamide has been demonstrated, current data is limited to in situ microparticles and may not extrapolate to other compositions [279].

Interest has also extended to hormone delivery systems. For instance, nanoparticle-based compositions containing progesterone have been developed using standard phase-inversion components [280], and prolonged-release glucocorticoid forms are being studied [281]. Unlike nanoparticles, testosterone-loaded phase-inversion systems showed favorable in vitro results without requiring specialized synthesis [282].

Ophthalmic applications are also emerging. Depot forms of dexamethasone [283] and fluocinolone acetonide [284] for intravitreal injection have shown promise, with histological analysis confirming absence of retinal damage despite concerns over localized pH reduction from matrix degradation.

Recent advancements include in situ depo-forms of dolutegravir for HIV [285], risperidone for psychiatric disorders [286], the anticoagulant dabigatran etexilate [287], and systems to enhance vaccine efficacy [288]. Additionally, a 2024 study by Lin X. et al. proposed the use of contrast-enhanced CT imaging to track implant morphology and drug release dynamics, offering a novel analytical tool previously unavailable in standard pharmaceutical analysis [289].

### 3.5. The Main Screening Parameters

For ISS that undergo phase inversion, the primary screening parameters shift from those describing the phase transition conditions to those related to the system’s properties before and after inversion.

In the liquid state, critical parameters such as pH, viscosity and other rheological characteristics, injectability (i.e., ease of injection through a needle), and surface wetting angle are assessed. Following phase inversion and implant formation, evaluations focus on mechanical properties, porosity and morphology, degradation, and active pharmaceutical ingredient (API) release. The inversion process is accompanied by solvent diffusion into the surrounding soft tissue, which can also be studied; in addition, the rate of phase inversion is an important characteristic.

Viscosity and rheological parameters are commonly measured using rotational viscometry: Brookfield viscometer [241] or a cone-plate rotational viscometer [290] at 25 °C (with some studies including measurements at physiological temperature). Analyses include plastic viscosity (following approximation by an appropriate rheological model for non-Newtonian fluids), yield strength, and the moduli of strength and elasticity.

Injectability is evaluated by measuring the force required to depress the syringe plunger to inject the system. In the study by Rein et al. [241], this test was performed using a syringe connected to an 18-gauge needle and a texture analyzer (TA.XT plus, Stable Micro Systems, Godalming, UK). A 1 mL sample was loaded into the syringe, and the texture analyzer’s upper probe was lowered at 1.0 mm/s under a constant force of 0.1 N until it contacted the syringe base. The force and work of injection were recorded over a 10 mm distance in triplicate.

The wetting angle was assessed in [291] by applying the ISS onto glass, paraffin, and agarose gel surfaces and measuring the contact angle using a goniometer (FTA 1000, First Ten Angstroms, Newark, CA, USA).

Agar gel models were employed to study phase transition parameters across studies. A typical model used a 0.6% agar gel in phosphate buffer (pH 6.8). In [292], an in vitro model of the periodontal pocket was simulated by drilling 6 mm diameter cylindrical holes into agar blocks. The size of the matrix formed at the agar interface was monitored over time under a stereomicroscope to study the rate of matrix formation, with control points at 0.4 and 24 h [293].

The same agar model was used to study solvent diffusion. Wells were filled with 150 µL of ISS stained with water-soluble amaranth dye (0.1 g/10 mL). The dye migration was documented under a stereomicroscope, and diffusion distance over time was measured in three replicates to calculate solvent diffusion rates.

To confirm in situ phase inversion, samples were injected into phosphate buffer (pH 6.8). In [291], 1 mL of sample in a 3 mL syringe was injected through an 18-gauge needle into 5 mL of buffer in a glass tube. Photographs were taken at 0, 5, 15, 30, and 60 min to monitor the formation of an opaque matrix. For improved in vitro/in vivo correlation, some authors recommended degassing and preheating the buffer to physiological temperature [253].

Post-formation, the mechanical properties of ISS matrices are analyzed. In [292], hardness and elasticity/plasticity were measured using a texture analyzer (TA.XT plus, Stable Micro Systems Ltd., Godalming, UK) in a 6 mm diameter agarose well mimicking a periodontal pocket (n = 6). After placing 200 µL of the sample and allowing complete matrix formation (typically 5–72 h), a 3 mm stainless-steel probe was driven into the matrix at 0.5 mm/s. The force–displacement curve was recorded, with the probe held for 60 s at a penetration depth of 1.5 mm, then withdrawn at 10 mm/s. The maximum force upon penetration and the force remaining after 60 s were used to compute the elasticity/plasticity ratio: higher values indicated greater elasticity.

The morphology of the formed implants is typically studied using scanning electron microscopy (SEM) or radiographic techniques [241]. In the study by Rein et al., matrices were incubated in phosphate buffer (pH 6.8) for seven days, washed with 200 mL distilled water, lyophilized, and stored in a desiccator for one week before gold-coating. SEM imaging was conducted at 15 kV to evaluate surface and cross-sectional morphology.

The same study also proposed using X-ray imaging and X-ray tomographic microscopy to examine implant morphology after identical sample preparation.

API release from phase-sensitive ISS has been investigated via equilibrium dialysis through membranes [241] or release into a dissolution medium using standard “Rotating Basket” or “Rotating Paddle” apparatuses at 37 °C and 50 rpm in phosphate buffer (100 mL). Although this method is often critiqued for limited physiological relevance, it is still valid for estimating API release into biological fluids, which in the case of dental dosage forms is equivalent to saliva. API diffusion with the solvent and release upon matrix erosion occurs into soft tissues, which existing in vitro tests fail to replicate adequately [294].

The in vitro degradation of the forming matrix in studies [291,292] was evaluated by measuring weight loss of the system after the drug release test. The initial weight of the sample and after the release test after 14 days was recorded and calculated (n = 3) as follows

% mass degradation = Wo − WtWo × 100

where

Wo—initial sample weight,

Wt—the weight of the remaining sample at a given point in time.

An amount of 1 g ISS sample was immersed in 10 mL of buffer solution (pH 6.8) and then incubated at 37 °C while shaking at 50 rpm. After 7 days, the ISS matrix was dried at 65 °C.

In summary, a review of the available methods and protocols for evaluating phase-sensitive ISS underscores the critical need for the development of physiologically relevant in vitro models that mimic soft tissues or target administration sites. Agar gels prepared at varying concentrations in phosphate buffer offer an optimal medium for such models, as they simulate soft tissue density and osmotic properties. Currently, such models have primarily been applied to assess periodontal pocket implants. However, the development of analogous models for subcutaneous adipose tissue or alveolar sockets appears highly promising for routine screening to optimize ISS composition.

Moreover, the need for relevant in vitro methods extends beyond diffusion and phase inversion rate studies. Present approaches to evaluating API release from phase- inversion systems lack physiological relevance. Adaptation of agar-based methods, similar to classical agar diffusion assays used for soft dosage forms, could provide more accurate assessments of release behavior [294].

## 4. Discussion

Despite growing interest from the global scientific community, the development and clinical implementation of phase-inversion systems are hindered by a range of technological and social challenges. Table 4 summarizes recent studies that highlight these issues.

### 4.1. Problems of Research Harmonization and Standardization

From the perspective of structural and mechanical properties, most researchers investigating in situ-forming implants have focused on pre-transition solution viscosity and injectability [240,241,242,246,254,256,258,265,270,271,290,291,292,293]. However, relatively few studies examined the visco-plastic or solid state of the formed implants and their structural characteristics [243,246,248,254,270]. For example, Siepmann F. and colleagues [243,246] studied the time-dependent adhesion and mechanical strength of PLGA-based implants using a texture analyzer. More recently, Phaechamud T.’s group [254] conducted similar investigations on Eudragit^®^-based formulations. These studies also demonstrated that the consistency and mechanical strength of the forming implant depended not only on the type of matrix-forming agent but also on the plasticizers incorporated into the composition [243].

Data on gelation time, active substance release, and degradation remain scarce and often contradictory. Degradation studies have primarily been conducted in vitro, limiting the ability to extrapolate results to human systems [240,241,242,246,254,256,258,265,270,271,290,291,292,293,294]. To date, no standardized methodology or model has been established to evaluate degradation. Gelation time was often omitted or unclearly reported in many studies [241,256], and published ISS photographs were frequently insufficient for accurate interpretation [241,292]. Drug release kinetics tend to be more predictable, typically following biphasic or triphasic release patterns depending on the polymer content [266] and matrix formulation [270,292]. Generally, release patterns follow Fick’s diffusion principles [292].

The variety of APIs tested in ISS remains limited. As has been shown in Table 4, most studies involved doxycycline hyclate [240,242,254,271,292], likely due to the success of Atridox^®^. However, this trend restricts broader ISS development.

Only a few studies have assessed ISS cytotoxicity [256,270,271]. Consequently, the potential for adverse reactions to individual components remains a concern, possibly leading to misleading efficacy and safety results—a critical issue for researchers.

In summary, research in this area is poorly harmonized, with substantial variation in equipment, methods, and experimental design. Future investigations are advised to emphasize a systematic evaluation of matrix-forming agents and excipients to ensure both mechanical integrity and safety of the final dosage form.

### 4.2. Regulatory Hurdles

Regulatory challenges represent a major obstacle for the advancement of ISS technologies. For instance, several commonly used matrix-formers and solvents have not yet received FDA approval [29,30,31,32,33]. Regulatory approval by the FDA [29] and inclusion in the pharmacopeias of various countries [30,31,32,33] serve as important incentives for pharmaceutical development. Toxicity concerns, local irritation, and environmental safety issues have been identified as major factors hindering the scientific advancement and clinical translation of phase-inversion ISS. As a potential solution, the use of environmentally safe materials already investigated as excipients in ISS (e.g., triacetin) could be explored. Alternatively, other green solvents, as well as biocompatible and biodegradable polymers, could be considered as promising candidates for the development of next-generation ISS. Beyond potentially improving patient and professional compliance, this approach aligns with current trends in pharmaceutical development that prioritize both human safety and environmental responsibility. However, even approved components often face legal constraints related to patent protection. For example, many formulations and technologies (e.g., PLGA-PEG + chlorhexidine) have been patent-protected for 17 years [295], and new developments using this system emerged shortly after the patent expired [296]. With the growing popularity of FluidCrystal^®^ technology, new patents involving phospholipids, spen, and ethanol are currently being registered [297], potentially opening new ways for ISS innovation.

### 4.3. Scale-Up Limitations

Transitioning from in vitro research to commercial-scale production involves not only further testing and regulatory review, but also industrial-scale technology transfer. Beyond core manufacturing steps, sterilization remains a critical challenge in polymer-based ISS development, as it may alter the drug’s pharmacological and technological properties [298]. While additional protective excipients may offer partial solutions, the limited understanding of phase-inversion systems necessitates thorough compatibility testing. Additionally, post-sterilization quality control [298] is essential to determine the acceptability of specific sterilization methods, underscoring the urgent need for standardized ISS quality assessment protocols.

### 4.4. The Problem of Patient Acceptance

Patient acceptance plays a crucial role in therapeutic adherence, particularly for chronic disease management. Although reduced dosing frequency can enhance compliance, several clinical trials have noted a higher incidence of adverse effects with ISS compared to conventional prolonged-release dosage forms [200]. Further research is crucial to elucidate the factors contributing to the increased incidence of adverse reactions observed with some ISS formulations, and to develop strategies for their mitigation or elimination. Therefore, the availability of registered medications is not the sole obstacle to the wider adoption of ISS in clinical practice.

### 4.5. Future Directions and Prospects

Several factors can contribute to the development of phase-inversion ISS. First, the establishment of a standardized term for such systems would improve indexing, enhance visibility, and clearly distinguish them from other sol–gel systems triggered by different mechanisms. Secondly, the development of standardized ISS quality control methods would enhance reproducibility, enable identification of potential improvements, and support scalability. Furthermore, we contend that comprehensive screening of co-formulation components is essential to develop non-toxic dosage forms with a minimized risk of adverse reactions.

Another promising avenue lies in the application of machine learning and artificial intelligence. However, the limited awareness of phase-inversion ISS, the restricted range of drugs employed, and the absence of standardized terminology and methodologies present significant challenges. Researchers often encounter limitations due to the scarcity of readily accessible information regarding both excipients and optimal combinations, particularly concerning APIs as well as comprehensive comparative analyses of formulations. Consequently, the development of specialized software or neural networks could streamline the search of existing databases and expedite compound screening processes.

Phase-inversion ISS could also be integrated with other advanced delivery systems, such as nanoparticles. Current research has explored combinations of phase-inversion matrices with thermosensitive polymers, which are better known to the global scientific community [88]. Furthermore, some of the polymers used in these systems have been studied in conjunction with rare earth metal nanoparticles. PCL is notable for providing stability to cerium dioxide nanoparticles [299]. Furthermore, PCL, polylactic acid, and PLGA have all found applications in the fabrication of biodegradable microneedles [300,301], although their joint application in ISS requires further investigation.

## 5. Conclusions

Although phase-inversion systems were among the earliest in situ-forming technologies, they have only gained significant attention in the past five years. This renewed interest likely reflects the complexity of requirements for system components, such as physical criteria (e.g., viscosity, mechanical strength), high biocompatibility standards, and the absence of any adverse reactions.

Multiple challenges hinder the advancement of these systems: the ambiguous toxicity profiles of many solvents; the biocompatibility of matrix-forming agents has a negative side—the risk of local disorders due to the influence of their degradation products; the customizable yet sensitive morphology and degradation profiles of the implants; and the intricate release kinetics that limit the range of drugs that could be introduced into the composition. Additionally, regulatory, manufacturing, quality control, and patient adherence barriers continue to limit broader adoption.

Despite these challenges, phase-inversion systems possess considerable potential, particularly if current obstacles can be overcome. The aim of this review, as of the first of the kind, was to highlight the defining characteristics, existing limitations, and promising future directions for phase-inversion drug delivery systems.

## Figures and Tables

**Table 1:** Current status of the main matrix-formers of phase-inversion systems.

	Availability Data	FDA [29]	USP [30]	EP [31]	JP [32]	EAEU Pharmacopeia [33]
Matrix Former						
lactic acid homopolymer		Approved	Mentioned	No data	Mentioned	Mentioned
polycaprolactone		No data	No data	No data	Mentioned	No data
*N*-lauroyl-L-alanine methyl ether		No data	No data	No data	No data	No data
sucrose acetate isobutyrate		Approved	No data	No data	No data	No data
borneol		Approved	No data	No data	Mentioned	Mentioned
shellac		Approved	Mentioned	Mentioned	Mentioned	No data
PLGA		Approved	No data	No data	Mentioned	No data

**Table 2:** Current status of the main solvents of phase-inversion systems.

	Availability Data	FDA [29]	USP [30]	EP [31]	JP [32]	EAEU Pharmacopeia [33]
Solvent						
*N*-methylpyrrolidone		Approved	Mentioned	Mentioned	Mentioned	Mentioned
dimethyl sulfoxide		Approved	No data	Mentioned	Mentioned	Mentioned
benzyl benzoate		No data	No data	Mentioned	Mentioned	No data
benzyl alcohol		Approved	No data	Mentioned	Mentioned	No data
triacetin		Approved	No data	Mentioned	Mentioned	Mentioned
triethyl citrate		Approved	Mentioned	Mentioned	Mentioned	No data
glycofurol		Approved	No data	No data	No data	No data
ethyl lactate		Approved	No data	No data	No data	No data
propylene carbonate		Approved	Mentioned	No data	No data	No data

**Table 3:** Highlights of clinical studies on phase-inversion systems («-» means there is no data).

Technology	API	Comparator Drug	Drug Administration	Blood Plasma Concentrations, ng/mL or Population Pharmacokinetic Model Data	Side Effects	Efficiency	Phase	Study Type	The Number of Participants	Date	Sources
Atrigel^®^	BPN	No comparison drug was available	Subcutaneous injection	-	-	-	1	A multicenter, open-label, single ascending dose study	48	2012–2013	[202]
		No comparison drug was available	Subcutaneous injection	-	-	-	1	An open-label, single-center, first-in-human study	18	2010–2011	[203]
		No comparison drug was available	Subcutaneous injection	-	-	-	1	A single-center, randomized, open-label, single-dose study	47	2015–2016	[204]
		No comparison drug was available	Subcutaneous injection	After the first injection: 2; before the second injection: 1.8–1.9; After the second injection: 3	64.1%; mostly constipation (30.8%), injection site reactions (79.5%)	therapeutic concentration was maintained for 28 days	2	A multiple-dose, single-center study	39	2013–2014	[205,206]
		No comparison drug was available	Subcutaneous injection	-	-	-	2	An open-label, multicenter, multiple dose study	124	2012–2014	[207]
		No comparison drug was available	Subcutaneous injection	After the first injection: 2; monthly injection: 5–10	-	-	3	An open-label, long-term study	775	2015–2017	[208,209]
		Placebo	Subcutaneous injection	-	For 1–6 months: 62.5–76.8% For 7–12 months: 38.1–58.0% Serious side effects: 2.7–4.4%	the percentage of participants abstinent from opioids: For 6 months: 65.5–66.3% For 12 months: 47.1–53.9%	3	An open-label, multicenter study	669 for safety study; 901 for efficiency study	2015–2017	[208,210]
		Placebo	Subcutaneous injection	-	For 1–6 months: 72.5% For 7–12 months: 55.6% For 13–18 months: 31.8% Serious side effects: For 2–6 months: 0.0–0.9% For 7–12 months: 5.4–7.8% For 13–18 months: 6.6–7.8% Severe side effects: For 2–6 months: 0.7–1.6% For 7–12 months: 0.0–0.5% For 13–18 months: 0.3–0.6%	the percentage of participants abstinent from opioids: For 18 months: 80–92.7%	3	An open-label, multicenter study	208	2016–2017	[211,212]
		No comparison drug was available	Subcutaneous injection	-	Total number of adverse events: 91% Number of people with a treatment-related adverse event: 45% Total number of serious adverse events: 26% Number of people with a serious adverse event over the treatment period: 16%	Treatment retention: For 12 months: 75% For 6 months: 86%	3	An open-label, multicenter, single-arm study	100	2019–2021	[213,214]
Atrigel^®^	RPD	No comparison drug was available	Subcutaneous injection	-	-	-	1	A multicenter, randomized, open-label, single-dose study	44	2016	[215]
Atrigel^®^		No comparison drug was available	Subcutaneous injection	Oral risperidone model: (1) rate constant for the absorption: 3.64; (2) rate constant for conversion to 9-hydroxyrisperidone: 0.0990; (3) rate constant for elimination by other processes: 0.0344; (4) elimination rate constant for 9-hydroxyrisperidone: 0.0782; (5) volume of distribution of the central compartment: 63.8; Atrigel^®^RPD model: (1) rate constant for the rapid absorption: 0.0266; (2) rate constant for the slow absorption: 0.0185, (3) transit rate constant: 0.0247; (4) rate constant for elimination by other processes: 0.011; (5) rate constant for conversion to 9-hydroxyrisperidone: 0.102; (6) exchange rates between the central and the peripheral compartments of risperidone: 0.572 and 0.0114; (7) volume of distribution of the central compartment: 171;	The results of Monte Carlo simulation analyses suggest a potentially safer profile of Atrigel^®^RPD compared to oral risperidone in relation to any extrapyramidal symptoms	-	2	An open-label, multiple ascending dose study	45	2012–2013	[216,217]
Atrigel^®^		Placebo	Subcutaneous injection	-	(1) Any side effects: 90 mg: 70.4% 120 mg: 77.8% Placebo: 68.6% (2) Any serious side effects: 90 mg: -% 120 mg: 0.9% Placebo: 0.9% (3) Any extrapyramidal symptoms: 90 mg: 7.8% 120 mg: 11.1% Placebo: 9.3%	Both Atrigel^®^RPD groups showed significant improvement with respect to positive symptoms. A significant improvement with respect to negative symptoms was found only in the 120 mg group	3	A randomized, double-blind, placebo-controlled, multicenter study	354	2014	[218,219]
Atrigel^®^		Placebo	Subcutaneous injection	-	A total of 73.4% of participants reported 1 or more side effects. Only 4.8% of participants reported one or more severe side effects. The most common side effects, associated with Atrigel^®^RPD, were injection-site pain (12.8%), weight increase (11.4%) and akathisia (5.8%).	Mean changes on the Positive and Negative Syndrome Scale: (1) the Positive Scale score: Placebo: −7.8 90 mg: −3.7 120 mg −3.7 (among rollover participants); Among de novo participants mean changes were −1.3; (2) the Negative Scale score: Placebo: −4.0 90 mg: −4.1 120 mg −0.9 (among rollover participants); Among de novo participants mean changes were −1.3; (3) the General Psychopathology Scale score: Placebo: −8.3 90 mg: −4.7 120 mg −6.4 (among rollover participants); Among de novo participants mean changes were −0.4;	3	An open-label, long-term safety and tolerability study	500	2014–2016	[220,221]
Atrigel^®^	ADX	Scaling and root planning; Metronidazole	-	-	-	-	2	A randomized interventional study	146	2003–2009	[222]
Atrigel^®^	LPA	No comparison drug was available	Subcutaneous injections	-	The rate of adverse events was 1.41%. The most common adverse events were asthenia, headache, arterial hypertension and nausea.	Average plasma PSA level decreased by 81.7% Average plasma testosterone level decreased by 88.0%; target testosterone level was achieved in 88.0–97.0% of patients	-	A prospective, multicenter, observational study	645	2013–2016	[223,224]
		No comparison drug was available	Subcutaneous injections	-	The incidence of side effects was 7.7%. The most common side effects were sexual impotence (6.2%) and hot flashes (2.5%). Severe toxicity was observed in 0.9% of cases	Mean plasma PSA levels decreased by 99.7% after 12 months	-	A retrospective study	932	2007–2018	[182]
		No comparison drug was available	Subcutaneous injections	-	75.5% of patients experienced at least one side effect. For example, the most common side effects were PSA level increase (17%), cough (9.4%) and hot flashes (7.5%). Severe toxicity was observed in 0.9% of cases	Average plasma PSA level decreased by 68.5–81.5% Average plasma testosterone level decreased by 51.4–80.0%;	4	An interventional study	107	2017–2019	[181,225]
BEPO^®^	CLX	Bupivacaine Hydrochloride	-	-	-	-	2	A randomized, single-blind, active-control, parallel group study	20	2018–2020	[226]
		Bupivacaine Hydrochloride+Acetaminophen+Methocarbamol	-	-	-	-	3	A multicenter, randomized, double-blind, parallel group study	151	2022–2024	[227]
		-	-	-	-	-	2–3	An open-label safety study	100	2025–2027	[228]
FluidCrystal^®^	BPN	Sublingual tablet Subutex^®^, infusion solution Temgesic^®^	Subcutaneous injection	C (trough): (1) 24 h after dosing for infusion solution: 0.042 (2) Sublingual tablet (2.1) First dose 8 mg: 0.52 16 mg: 1.1 24 mg: 1.47 (2.2) Seventh dose 8 mg: 1.52 16 mg: 3.81 24 mg: 4.11 (3) FluidCrystal^®^ (3.1) weekly 16 mg: First dose 0.29 Fourth dose 0.42 (3.2) monthly 64 mg: 0.14 96 mg: 0.15 128 mg: 0.27 192 mg: 0.39	The most commonly reported side effects after FluidCrystal^®^ BPN administration were nausea (63%) and dizziness (54%). The number of side effects was smallest for the FluidCrystal^®^ formulations, followed by sublingual tablets and infusion solution	-	1	An open label, randomized controlled study	87 for safety study, and 75 subjects for pharmacokinetics study	2014–2016	[197]
		No comparison drug was available	Subcutaneous injection	C trough: 24 mg: First dose 1.81 Second dose 2.29 32 mg: First dose 2.24 Second dose 3.05	A total of 64% of participants with 24 mg and 96% of participants with 32 mg dose experienced 1 or more side effects. Adverse events suspected to be related to FluidCrystal^®^ BPN were reported by 57.4%, including constipation (19%) and injection-site pain (9%), with most rated as mild severity.	Opioid withdrawal was completely suppressed on day 1 after FluidCrystal^®^ BPN injection and remained suppressed for the study duration	2	A multi-site, randomized, double-blind, repeat-dose study	47	2015–2016	[229]
		No comparison drug was available	Subcutaneous injection	-	In total, 56.4% of side effects were mild or moderate in intensity: injection-site pain (15.4%), swelling (11.9%), erythema (9.3%) and headache (7.9%). 6.6% of side effects were severe intensity	For new-to-treatment participants, the composite of illicit opioid-negative urine samples and self-reports was 63.0%. For other participants, the percentage negative for illicit opioids was 82.8%	3	An open-label, multicenter study	228	2015–2017	[230,231]
		Placebo+buprenorphine/naloxone	Subcutaneous injection	-	The most common adverse events (regardless of study medication) were injection-site pain (8.4%), headache (7.7%), constipation (7.5%) and nausea (7.5%). All injection-site adverse events were mild (73.9%) or moderate (26.1%) in intensity. In total, 4.2% of patients experienced at least 1 nonfatal serious adverse event	Treatment difference between increased from 5.9% to 8.5% relative to the comparison group during 3 months	3	A randomized, double-blind, active-controlled, parallel group, multicenter trial	428	2015–2016	[232,233]
	OCT	Long-acting octreotide	Subcutaneous injection	Ctrough FluidCrystal^®^ OCT: 10 mg: 0.24 20 mg: 0.52–0.53 30 mg: 0.82–0.86 Ctrough long acting octreotide 30 mg: 0.61	(1) Total number of side effects: 10 mg: 93.3–100% 20 mg: 85.7–100% 30 mg: 100% long acting octreotide 30 mg: 71.4% (2) Diarrhea: 10 mg: 80–88.2% 20 mg: 78.6–81.3% 30 mg: 76.5–80% long acting octreotide 30 mg: 35.7% (3) Headache: 10 mg: 39.5–41.9% 20 mg: 39.4–44.2% 30 mg: 41.2–53.3% long acting octreotide 30 mg: 49.2% (4) Abdominal pain: 10 mg: 33.3–41.2% 20 mg: 28.6–37.5% 30 mg: 26.7–52.9% long acting octreotide 30 mg: 34.4% (5) Injection site pain: 10 mg: 11.8–20% 20 mg: 21.4–25% 30 mg: 21.4–29.4% long acting octreotide 30 mg: 22.1%	Maximum inhibition of IGF-1 concentration for FluidCrystal^®^ OCT: (1) First injection 10 mg: 39.5–41.9% 20 mg: 39.4–44.2% 30 mg: 42.8–43.8% maximum inhibition of IGF-1 concentration for long acting octreotide 30 mg: 35.7% (2) Second injection 10 mg: 36.3–37.2% 20 mg: 37.5–40.0% 30 mg: 39.1–36.2% maximum inhibition of IGF-1 concentration for long acting octreotide 30 mg: 41.2% (3) Third injection 10 mg: 36.3–35.6% 20 mg: 33.5–45.4% 30 mg: 39.3–44.8% maximum inhibition of IGF-1 concentration for long acting octreotide 30 mg: 41.4%	1	A randomized, open-label parallel group study	122	-	[200]
		Placebo	Subcutaneous injection	-	-	-	2–3	A randomized, placebo-controlled, double-blind, multicenter trial	71	2022–2027	[234]
		Long-acting octreotide	Subcutaneous injection	(1) Acromegaly Ctrough FluidCrystal^®^ OCT (day 56): 10 mg: 1.0 20 mg: 1.0 Ctrough long acting octreotide (day 28): 10 mg: 0.2 30 mg: 1.2 (2) neuroendocrine tumors Ctrough FluidCrystal^®^ OCT (day 56): 10 mg: 1.3 20 mg: 1.7 Ctrough long acting octreotide (day 28): 20 mg: 0.9 30 mg: 1.3	Total number of side effects: 50–67%. The most commonly reported side effects: diarrhea (25%), injection site pain (25%), nausea (8.3%) and headache (16.7%)	(1) Acromegaly: IGF-1 maintenance (2) Neuroendocrine tumors: Maintaining symptom control or improving symptoms of carcinoid.	2	An open-label, multicenter, randomized study	12	2015–2016	[235,236]
	LPA	Eligard^®^	Subcutaneous injection	-	-	-	2	An open-label, multicenter, randomized study	51	2014–2016	[237]
	PST	Pasireotide immediate release and pasireotide long-acting release	Subcutaneous injection	Ctrough (1) pasireotide immediate release: 600 μg: 1.12 900 μg: 2.06 (2) pasireotide long-acting release 60 mg: 7.85 (3) FluidCrystal^®^ PST thigh: 5 mg: 0.30 10 mg: 0.51 20 mg: 0.85 40 mg: 2.17 80 mg: 4.09 buttock: 20 mg: 0.96	(1) Pasireotide immediate release (900 μg): Total number of side effects: 100% Injection site pain: 0% Diarrhea: 60.0% Nausea: 50.0% (2) pasireotide long-acting release (60 mg): Total number of side effects: 80.0% Injection site pain: 10.0% Diarrhea: 40.0% Nausea: 40.0% (3) FluidCrystal^®^ PST thigh: 5 mg: Total number of side effects: 50.0% Injection site pain: 16.7% Diarrhea: 0% Nausea: 0% 10 mg: Total number of side effects: 83.3% Injection site pain: 25.0% Diarrhea: 0% Nausea: 0% 20 mg: Total number of side effects: 91.7% Injection site pain: 50.0% Diarrhea: 0% Nausea: 0% 40 mg: Total number of side effects: 100% Injection site pain: 41.7% Diarrhea: 25.0% Nausea: 16.7% 80 mg: Total number of side effects: 100% Injection site pain: 66.7% Diarrhea: 100% Nausea: 16.7% buttock: 20 mg: Total number of side effects: 50.0% Injection site pain: 14.3% Diarrhea: 0% Nausea: 7.1%	Maximum inhibition of IGF-1 concentration relative to baseline: (1) pasireotide immediate release: 600 μg: −24.41 900 μg: −52.40 (2) pasireotide long-acting release 60 mg: −46.76 (3) FluidCrystal^®^ PST thigh: 5 mg: −24.64 10 mg: −36.32 20 mg: −27.50 40 mg: −50.67 80 mg: −58.87 buttock: 20 mg: −35.22	1	A randomized, open-label study	94	-	[201]

**Table 4:** Main characteristics of phase-inversion systems («-» means there is no data).

Matrix	Solvent	Degradability	Gelation Time	Burst Release Tendency	Compatibility with Drugs	Cytotoxicity	The Force of Syringeability, N	Sources
Shellac	NMP	41.18–63.42%	The speed of this transformation was as follows: DMSO > NMP > 2-Pyrrolidone > eutectic mixture	54.01% (168 h)	doxycycline hyclate		29.63–40.14	[240]
		75.00%	-	45.00% (15 h); 68.00% (168 h)	doxycycline hyclate	-		[293]
	DMSO	32.40–62.49%	-	61.72% (168 h)	doxycycline hyclate	-	31.00–40.14	[240]
		71.00%	-	60.00% (15 h); 78% (168 h)	doxycycline hyclate	-		[293]
	2-Pyrrolidone	80.05–99.98%	-	43.91% (168 h)	doxycycline hyclate	-	34.77–48.66	[240]
		92.00%	-	20.00% (15 h); 82% (168 h)	doxycycline hyclate	-		[293]
	Eutectic mixture of menthol and camphor	64.81–92.90%	-	33.67% (168 h)	doxycycline hyclate	-	86.81 ± 8.26	[240]
β-cyclodextrin	DMSO	78.54–93.32%	5–140 min	23.60–78.60% (4 h)	meloxicam	-	5.00–20.00	[240]
Eudragit^®^	NMP	-	Immediately; 0.5 min	55–90% (16.67 h)	doxycycline hyclate	-	3.00–66.00	[242]
		-	0.5 min	-	doxycycline hyclate	-	F remaining/F max deformation ≤ 0.1	[254]
		59.65–63.12%	1 min	dialysis method: 20–35% (16.67 h) membrane-less method:65–78% (16.67 h)	doxycycline hyclate	-	24.72–40.37	[291]
	2-Pyrrolidone	-	1 min	64.18–83.60% (10 h); 75.36–87.94% (24 h)	doxycycline hyclate	-	-	[251]
	monopropylene glycol	100%	1 min	38.00–50.00% (24 h)	levofloxacin hydrochloride	-	1.02–30.87	[290]
PLGA	NMP	-	-	25.00–50.00% (24 h)	doxycycline hyclate	-	F remaining/F max deformation ≤0.2	[243]
		-	-	-	chlorhexidine dihydrochloride; ibuprofen	-	0.22–0.27	[248]
		-	Immediately	71.00–75.00% (24 h)	cannabidiol	The signs of toxicity *in ovo*	Predominantly inadequate syringeability	[256]
	DMSO	-	Immediately	26.00–61.00% (24 h)	cannabidiol	no	Predominantly inadequate syringeability	[256]
Poly (DL-lactide)	NMP	-	Immediately	32.80- 58.45% (24 h)	tenoxicam	Moderate inflammatory cellular infiltration after 7 days in vivo	13.50–127.00	[270]
Benzoin	DMSO	-	1 min	23.00% (24 h)	vancomycin hydrochloride	-	0.40–0.60	[246]
Propolis	DMSO	-	1 min	40.00% (24 h)	vancomycin hydrochloride	-	0.10–0.13	[246]
Rosin	DMSO	-	1 min	58.00% (24 h)	vancomycin hydrochloride	-	0.33–0.45	[246]
Borneol	DMSO	58–75%	5 min	45.00% (24 h)	vancomycin hydrochloride	-	-	[250]
Lauric acid	NMP	-	1 min	-	clove oil	-	0.783–0.936	[265]
Poly (isosorbide succinate)	NMP	-	-	17.00% (24 h)	doxycycline hyclate	No cytotoxicity was observed in the experiment with fibroblasts and aortic smooth muscle cells	-	[271]
cholesterol	NMP, menthol and benzyl benzoate	70.78–99.50%	10 min	70.00–80.00% (16.67 h)	doxycycline hyclate	-	1.08–2.03	[292]

## Data Availability

No new data were created or analyzed in this study. Data sharing is not applicable to this article.
